# America’s Great Tonic

**Published:** 1918-05

**Authors:** John Price Jones

**Affiliations:** Assistant Director, in Charge, Press Bureau. Liberty Loan Committee, Second Federal Reserve District, 120 Broadway, New York


### America's Great Tonic.

JOHN PRICE JONES,

Assistant Director, in Charge, Press Bureau.

Liberty Loan Committee, Second Federal Reserve District,

120 Broadway, New York.

(Written exclusively for "Buffalo Medical Journal.")

The Liberty Loans might be likened to great tonics, refreshing and building up a combative body of huge proportions. It is this giant, panoplied in all the safeguards and offensive instruments that modern imagination has conceived, who is to receive its third tonic, in the shape of another loan, on April 6.

The necessity for a further upbuilding of this giant is apparent if the ideals that America was nurtured in and by which she is claiming the right of existence are to endure. Abraham Lincoln in immortal words said long ago that this country was "dedicated to the proposition that all men are created equal." That ideal is one that America has kept constantly before her eyes, and it is because the Hun has sought to make of himself a conscienceless Obermensch or superman, destroying the values of all creative communities about him, subduing his own dependencies to the class of speechless menials and generally abrogating to himself in a selfish manner the tools of power that the Republic is in this war. She is in it to the finish because of her unalterable opposition to all that is destructive in civilizaton. It is the duty, therefore, of every person of sincere and honorable ideals in this country to forward the war, to bring it to a speedy and successful conclusion.

That great object may be obtained if the gigantic military machine fighting for truth and right and America is properly strengthened and kept at formidable proportions. It is time for the third tonic; martial days disastrous in their consequences to the enemy loom ahead and our chivalric giant must be in exceptional condition to meet them. The Third Liberty Loan is a tonic which is bound to prove a decisive factor in hardening the unconquerable qualities of America's loyal giant. It is the duty of every man to aid in the preparation of this great tonic.

---

**Prosthetic Use of Hard Rubber.** Delbet and Girode, recommend hard rubber cuffs in extensive loss of bone as in the humerus and similar appliances to afford external support.

